# Supplementary material for: Specific patterns of neural activity in the hippocampus after massed or distributed spatial training
Source: Sci Rep. 2023 Aug 16;13:13357. doi: 10.1038/s41598-023-39882-0 (PMC10432541; doi:10.1038/s41598-023-39882-0)
Supplement: Supplementary file 1 — Supplementary Information. [file 41598_2023_39882_MOESM1_ESM.docx]

**Supplementary Material**

**Specific patterns of neural activity in the hippocampus after massed or distributed spatial training**

Eleonora Centofante^1,2^, Luca Fralleoni^1^, Carmen A. Lupascu^2^, Michele Migliore^2^, Arianna Rinaldi^1^, Andrea Mele^1^

^1^Department of Biology and Biotechnology ‘C. Darwin’- Centre for Research in Neurobiology ‘D.Bovet’, Sapienza University of Rome, Italy; ^2^Institute of Biophysics, National Research Council, Palermo, Italy;

**Matlab codes**

**Mata**

[r,c]=find(a>40);

mata=zeros(size(a);

for i=1:size(a,1)

for j=1:size(a,2)

dist=[];

if a(i,j)>40

mata(i,j)=2

for k=1:size(r,1)

if sqrt((r(k)-i)^2+(c(k)-j)^2)>0

dist=[dist sqrt((r(k)-i)^2+(c(k)-j)^2)];

end

end

i

j

a(i,j)

dist

size(find(dist<=5),2)

if size(find(dist<=5),2)>=5

mata(i,j)=size(find(dist<=5),2)

end

end

end

end

imagesc(mata)

**Matrixa**

function [mata,nra]=matrixa(a)

[r,c]=find(a>60);

nra=0;

mata=zeros(size(a));

for i=1:size(a,1)

for j=1:size(a,2)

dist=[];

if a(i,j)>40

mata(i,j)=2;

for k=1:size(r,1)

if sqrt((r(k)-i)^2+(c(k)-j)^2)>0

dist=[dist sqrt((r(k)-i)^2+(c(k)-j)^2)];

end

end

%i

%j

%a(i,j)

%dist

%size(find(dist<=5),2)

if size(find(dist<=5),2)>=3

mata(i,j)=size(find(dist<=5),2);

nra=nra+1;

end

end

end

end

end

**Histcut_23.m**

B=B_X_m;

[counts2 binCenters2] = hist(B(B(:)~=0), 20);

counts3=counts2/sum(counts2);

i=1;

while i<=size(binCenters2,2)

s=sum(counts3(1:i));

if s>=0.75

break;

end

i=i+1;

end

figure;imagesc(B,[0 121]);

hold on;

coordzone_B_X_m_23=[];

for k=1:size(B,1)

for j=1:size(B,2)

if B(k,j)>=23

**Pixels_m_23.m**

MATPIXSP=zeros(size(B_X_1));

MATPIXM=zeros(size(B_X_1));

for i=1:12

for j=1:12

[tf, index]=ismember([i j],coordzone_B_X_m_23,'rows');

if (tf==1)

MATPIXSP(j,i)=MATPIXSP(j,i)+1;

end

if not(isequal(B_X_m(j,i),0))

MATPIXM(j,i)=MATPIXM(j,i)+1;

End

figure;imagesc(MATPIXSPT)

figure;imagesc(MATPIXM)

hold on;

for k=1:12

for j=1:12

a=0;

if B_X_m(k,j)>=23

a=a+1;

end

%if MATPIXM(k,j)==a && a~=0

if MATPIXM(k,j)==a && a>=3

plot(j, k, 'r+', 'MarkerSize', 5, 'LineWidth', 3);

end

end

end

MATPIXSPT2=zeros(size(B_1_1));

for k=1:12

for j=1:12

a=0;

if B_X_m(k,j)>=23

a=a+1;

end

if MATPIXM(k,j)==a && a>=6 (spaced) a>=5 (massed) a>=13; 5; 6(control)

MATPIXSPT2(k,j)=a;

end

end

end

figure;imagesc(MATPIXSPT2)

**Common_23**

MATPIXSP10=zeros(size(B_X_1));

MATPIXM=zeros(size(B_X_1));

for i=1:12

for j=1:12

[tf, index]=ismember([i j],coordzone_B_X_m_23,'rows');

if (tf==1)

MATPIXSPX(j,i)=MATPIXSPX(j,i)+1;

if MATPIXSPT(j,i)==1

MATPIXSPX(j,i)=2;

end

end

end

end
